# Supplementary material for: Diagnostic Age, Age at Death and Stage Migration in Men Dying with or from Prostate Cancer in Denmark
Source: Diagnostics (Basel). 2022 May 19;12(5):1271. doi: 10.3390/diagnostics12051271 (PMC9140637; doi:10.3390/diagnostics12051271)
Supplement: Supplementary file 1 [file diagnostics-12-01271-s001.zip › diagnostics-1736933-supplementary.pdf]

Table S1. Temporal trends in age at diagnosis and death.

Stage	Age at diagnosis			Age at death		
	Overall	Prostate cancer-specific death	Other cause death	Overall	Prostate cancer-specific death	Other cause death
	Slope (95CI)	Slope (95CI)	Slope (95CI)	Slope (95CI)	Slope (95CI)	Slope (95CI)
Localized	-0.29*** (-0.16 – 0.07)	-0.10* (-0.20 – 0)	-0.39*** (-0.47 –0.31)	0.09** (0.03 – 0.15)	0.24*** (0.14 – 0.34)	0.03 (-0.06 – 0.11)
Locally advanced	0.03 (-0.07 – 0.13)	0.02 (-0.11 – 0.15)	0.01 (-0.13 – 0.16)	0.36*** (0.27 – 0.46)	0.33*** (0.20 – 0.45)	0.39*** (0.24 – 0.53)
Metastatic	0.02 (-0.08 – 0.12)	0.01 (-0,11 – 0.12)	0.05 (-0.15 – 0.24)	0.23*** (0.13 – 0.33)	0.18** (0.07 – 0.29)	0.36*** (0.16 – 0.56)
* p<0.05; **p<0.01; ***p<0.001; 95CI: 95% confidence interval						

Table S2. Formulas for linear regressions. X depicts calendar year of death and Y depicts either age at diagnosis or age at death.

Stage	Age at diagnosis			Age at death		
	Overall	Prostate cancer-specific death	Other cause death	Overall	Prostate cancer-specific death	Other cause death
All	Y=-0.12*X+309.13	Y=-0.02*X+116.20	Y=-0.26*X+600.52	Y=0.23*X-377.35	Y=0.26*X-445.80	Y=0.14-202.49
Localized	Y=-0.29*X+652.69	Y=-0.10*X+279.59	Y=-0.39*X+861.35	Y=0.09*X-104.36	Y=0.24*X-396.42	Y=0.03*X+29.17
Locally advanced	Y=0.03*X+12.15	Y=0.02*X+36.16	Y=0.01*X+47.06	Y=0.36*X-653.48	Y=0.33*X-577.45	Y=0.39*X-702.83
Metastatic	Y=0.02*X+25.34	Y=0.01*X+61.12	Y=0.05*X-15.84	Y=0.23*X-387.62	Y=0.18*X-285.35	Y=0.36*X-640.04

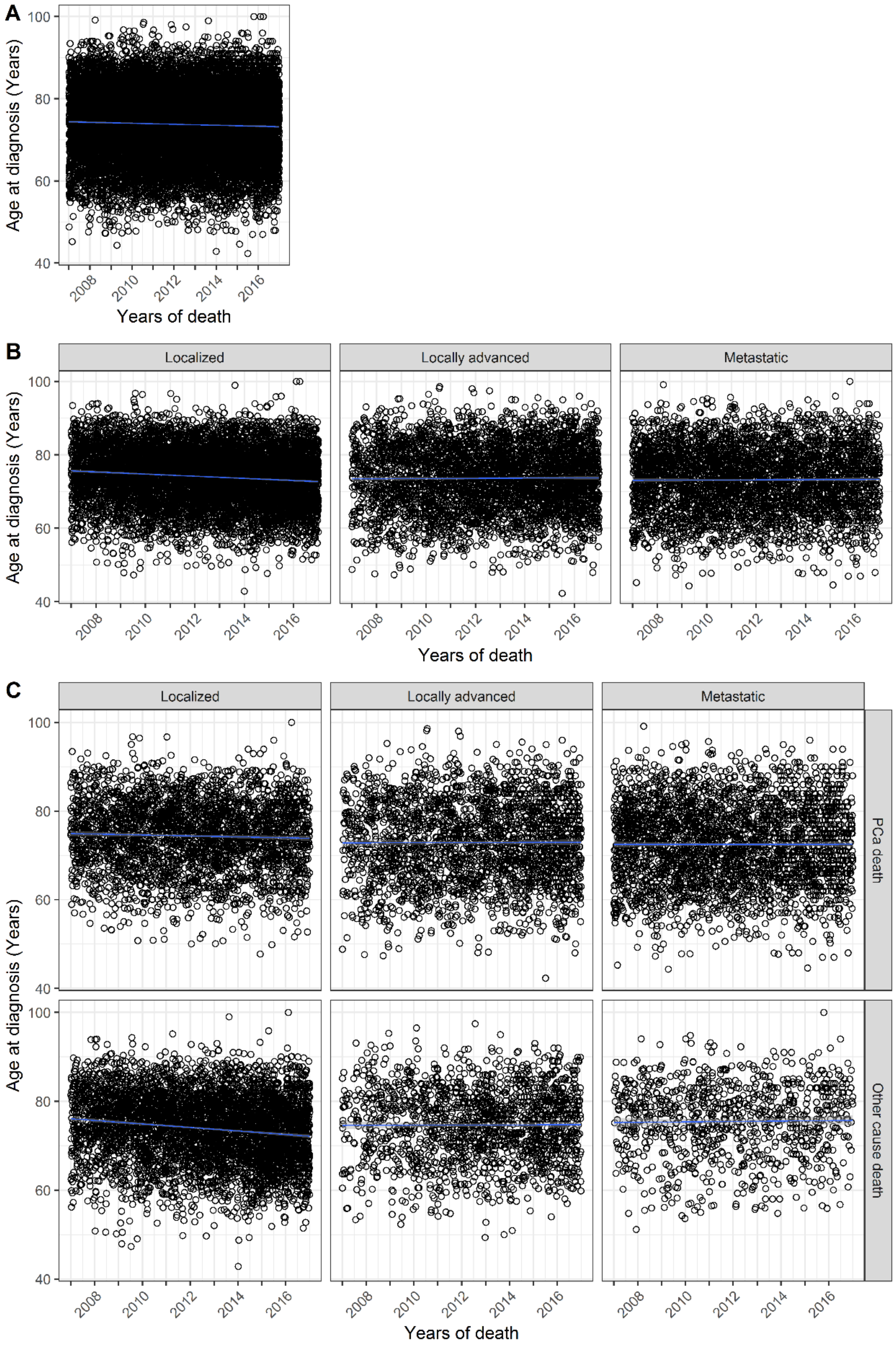

Figure S1. Scatterplot with linear regression of age at diagnosis for time of death. A) in the total cohort, B) stratified by stage at diagnosis and C) stratified by stage at diagnosis and type of death. Abbreviation: PCa= prostate cancer.

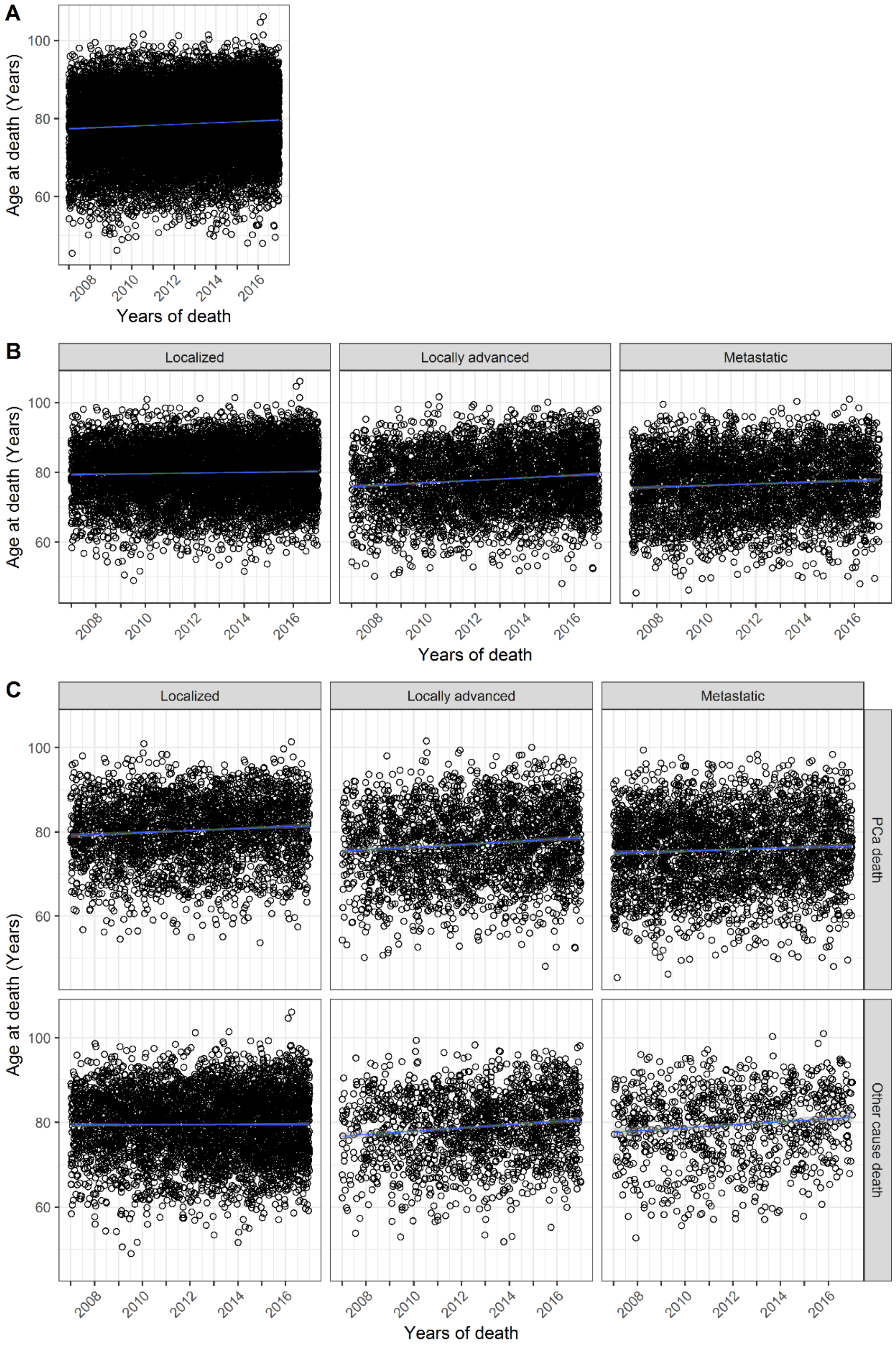

Figure S2. Scatterplot with linear regression of age at death for time of death. A) in the total cohort, B) stratified by stage at diagnosis and C) stratified by stage at diagnosis and type of death. Abbreviation: PCa= prostate cancer.
